# Prediction of activity-related energy expenditure under free-living conditions using accelerometer-derived physical activity

**DOI:** 10.1038/s41598-022-20639-0

**Published:** 2022-10-04

**Authors:** Stephanie Jeran, Astrid Steinbrecher, Verena Haas, Anja Mähler, Michael Boschmann, Klaas R. Westerterp, Boris A. Brühmann, Karen Steindorf, Tobias Pischon

**Affiliations:** 1 Molecular Epidemiology Research Group, Max Delbrück Center for Molecular Medicine (MDC) in the Helmholtz Association, Robert-Rössle-Straße 10, 13125 Berlin, Germany; 2 Division for Quality Management in Rehabilitative Care, Epidemiology and Statistics, German Statutory Pension Insurance Scheme, Berlin, Germany; 3 Department of Child and Adolescent Psychiatry, Charité-Universitätsmedizin Berlin, Corporate Member of Freie Universität Berlin, Humboldt-Universität zu Berlin, and Berlin Institute of Health, Berlin, Germany; 4 Experimental and Clinical Research Center, A Cooperation of Charité - Universitätsmedizin Berlin and Max Delbrück Center for Molecular Medicine, Berlin, Germany; 5 German Center for Cardiovascular Research (DZHK), Partner Site Berlin, Berlin, Germany; 6 Berlin Institute of Health (BIH), Berlin, Germany; 7 Nutrition and Translational Research in Metabolism (NUTRIM), Maastricht University Medical Centre, Maastricht, The Netherlands; 8 Institute of Medical Biometry and Statistics, Section of Health Care Research and Rehabilitation Research (SEVERA), Faculty of Medicine and Medical Center, University of Freiburg, Freiburg, Germany; 9 Division of Physical Activity, Prevention and Cancer, German Cancer Research Center (DKFZ), and National Center for Tumor Diseases (NCT), Heidelberg, Germany; 10 Charité –Universitätsmedizin Berlin, Berlin, Germany; 11 MDC/BIH Biobank, Max Delbrück Center for Molecular Medicine and Berlin Institute of Health, Berlin, Germany

**Keywords:** Epidemiology, Risk factors

## Abstract

The purpose of the study was to develop prediction models to estimate physical activity (PA)-related energy expenditure (AEE) based on accelerometry and additional variables in free-living adults. In 50 volunteers (20–69 years) PA was determined over 2 weeks using the hip-worn Actigraph GT3X + as vector magnitude (VM) counts/minute. AEE was calculated based on total daily EE (measured by doubly-labeled water), resting EE (indirect calorimetry), and diet-induced thermogenesis. Anthropometry, body composition, blood pressure, heart rate, fitness, sociodemographic and lifestyle factors, PA habits and food intake were assessed. Prediction models were developed by context-grouping of 75 variables, and within-group stepwise selection (stage I). All significant variables were jointly offered for second stepwise regression (stage II). Explained AEE variance was estimated based on variables remaining significant. Alternative scenarios with different availability of groups from stage I were simulated. When all 11 significant variables (selected in stage I) were jointly offered for stage II stepwise selection, the final model explained 70.7% of AEE variance and included VM-counts (33.8%), fat-free mass (26.7%), time in moderate PA + walking (6.4%) and carbohydrate intake (3.9%). Alternative scenarios explained 53.8–72.4% of AEE. In conclusion, accelerometer counts and fat-free mass explained most of variance in AEE. Prediction was further improved by PA information from questionnaires. These results may be used for AEE prediction in studies using accelerometry

## Introduction

Activity-related energy expenditure (AEE) is the component of total daily energy expenditure (TDEE) showing the largest within-person variance, and therefore plays an important role in body weight regulation and obesity research. AEE can be defined as the component of energy expenditure that is caused by “any bodily movement produced by skeletal muscles”^1^. In addition to physical activity (PA), individual factors such as age, sex, height or body composition are associated with AEE^2,3^.

Under laboratory conditions, AEE can be directly measured by indirect calorimetry in a respiration chamber, which is, however, not feasible for longer time periods under free-living conditions. Free-living AEE can be calculated as TDEE minus resting energy expenditure (REE) and diet-induced thermogenesis (DIT). The gold standard for measuring TDEE under free-living conditions is the doubly-labeled water (DLW) method, while REE can be determined by indirect calorimetry under fasting and resting conditions. However, in large-scale epidemiological studies these time-consuming and cost-intensive methods are not feasible. Therefore, AEE has been traditionally derived from PA information that was collected via questionnaires and linked to activity-specific energy costs (i.e. metabolic equivalent of task, MET values) to calculate an individual’s energy expenditure. Due to limitations of questionnaire-based assessment (e.g. subjective interpretation of duration and intensity of PA, memory issues), PA and thus energy expenditure may be prone to substantial under- or overestimation^4,5^.

Accelerometry provides convincing opportunities for PA recording and, subsequently, estimating AEE, and is now used in several large-scale epidemiological studies, such as the German National Cohort^6^ or the UK Biobank^7^. Small accelerometer devices are attached to specific body sites and objectively capture acceleration of body movements in up to three planes to provide information about frequency, intensity and duration of PA^8^. Analogue to questionnaire-derived PA information, the accelerometer output may be converted to energy expenditure estimates using device-specific algorithms, which were developed when performing standardized activities under controlled laboratory conditions^9–12^.

Although many (device-dependent) algorithms were available, it is unclear to what extent accelerometry-derived PA can explain the variance in AEE under free-living conditions, and to what extent other individual characteristics could improve this estimation of AEE. In a previous systematic review, we found a large heterogeneity across studies in the explained variance of free-living AEE estimated from accelerometry, ranging from 4 to 80%^13^. Importantly, inclusion of other predictors in addition to accelerometer output significantly increased the explained variance but a large heterogeneity remained across studies^13^.

The aim of this study was, therefore, to develop prediction models to estimate AEE in a free-living setting (i.e. DLW-derived) based on accelerometry-derived PA information and additional factors, and to clarify each factor’s impact on explaining AEE variance. In the context of practical use, different settings of additional factors were considered to investigate each factor’s potential of improving the AEE variance, and to provide opportunities for AEE prediction depending on predictors’ availability.

## Methods

### Study population

The ActivE-study was conducted between September 2012 and April 2014. Our sample size calculation revealed a total number of 49 participants for a given alpha level of 0.05, a power of 0.80, a partial correlation of 0.4 between accelerometer output and AEE, and four predictors included in the model. Stratified by sex (50:50), age (five 10-year age groups), and body mass index (BMI, 18.5–24.9, 25–29.9, > 30.0 kg·m^−2^), 50 volunteers were recruited via institutional mailing lists, advertisement in newspapers and local stores in Berlin and surrounding area (Supplementary Fig. S1). Inclusion criteria were: age 20–69 years, BMI 18.5–35.0 kg·m^−2^, German language skills, and everyday life conditions. Exclusion criteria were: pregnancy or lactation period, addiction to drugs or alcohol, cardiovascular disease, diabetes mellitus, instable thyroid disease, weight changes > 10 kg during previous six months, participation in weight reduction programs during previous three months, hemophilia, or intake of medication interfering with energy expenditure. In case of acute infections or vacation, the examination was postponed.

### Data collection

The study was performed in accordance with the relevant guidelines and regulations and the study protocol was approved by the local ethics committee of the Charité-Universitätsmedizin Berlin. All participants gave written informed consent prior to study entry and visited the study center at the first and last day of the 2-week examination period. At the first visit, we measured anthropometric parameters such as height (using stadiometer SECA 285, Hamburg, Germany), weight (using SECA mBCA 515 (Hamburg, Germany), BMI, hip and waist circumference (using measuring tape SECA 201, Hamburg, Germany), and fat mass and fat-free mass (FFM) (using bioelectrical impedance analysis (BIA) with SECA mBCA 515 and air-displacement plethysmography (ADP) with BODPOD® (Life Measurement, Inc., Concord, CA, USA). We measured blood pressure and resting heart rate (using hemodynamometer OMRON HEM 705 IT, Mannheim, Germany) and maximum handgrip strength (using Jamar Plus dynamometer, Patterson Medical, Bolingbrook, IL, USA), collected information on sociodemographic factors (age, sex, school education, professional training/qualification, occupation), lifestyle factors (smoking habits, alcohol consumption, PA by using IPAQ-Short^14^, sleep duration), medical history and current medication in a personal questionnaire-based interview, collected one basal urine sample for DLW analyses, and equipped each participant with an accelerometer. During the 2-week examination period, participants wore the accelerometer continuously 24 h per day (except for sauna, water-based activities or high contact sports) and documented non-wear times, collected DLW urine samples at five pre-specified time points, and completed a 7-day dietary record (197 common food items in conventional portion sizes) that was analyzed using software Optidiet (V5.0.2.010, GOE mbH, Linden, Germany). At the second visit, we collected the last DLW urine sample, measured REE, and participants completed the ‘Questionnaire on PA on previous 12 months’ (QUAP). All measurements followed standardized protocols.

To determine daily PA, we used the triaxial accelerometer ActiGraph GT3X + (ActiGraph TM, Pensacola, FL, USA) placed at the right hip, and the corresponding software ActiLife (versions 6.11.0–6.11.9; ActiGraph LLC, Fort Walton Beach, FL, USA). Acceleration raw data were recorded at 100 Hz sample rate using all three spatial axes with disabled ‘Idle sleep mode’. Along with the download, ActiLife converted the raw data into ‘counts’. We used an epoch length of one second to condense the data. Due to limited recording capability under the specified conditions, each participant switched to a provided pre-initialized second accelerometer for data recording in the second examination week. From the merged data set, we derived the following activity parameters as daily averages: Axis 1 counts per minute (cpm), Axis 2 cpm, Axis 3 cpm, vector magnitude (VM) cpm, steps per day and steps per minute. Using an adapted version of the ‘Freedson Adult VM3 (2011)’ algorithm^15^, we calculated the time spent in PA of vigorous (6167 cpm and above), moderate (2690–6166 cpm), and low (79–2689 cpm) intensity, as well as time spent inactive (0–78 cpm). Based on these parameters, we calculated total and relative time in low, moderate and vigorous PA. For analysis, activity parameters were arithmetically averaged across complete days. Incomplete days due to study center visits or substantial non-wear time during waking hours (> 8 h) were excluded (details in Supplementary Table S1).

To determine average TDEE, we used the DLW method over the 2-week examination period according to an adapted version of the Maastricht protocol^16^. The basal urine sample was collected at the midmorning at the first study center visit (day 1). Each participant took his individually weighed DLW dose before going to bed on that day. The first enriched urine sample was collected from the second voiding at the next morning (day 2). Further urine samples were collected on day 2 from a random voiding in the evening, on day 9 from the second voiding in the morning and from a random voiding in the evening, on day 14 from a random voiding in the evening, and on day 15 from the second voiding in the morning. The DLW isotope concentration of each urine sample was measured in duplicates using an isotope-ratio mass spectrometer. The difference in isotopes’ elimination rates revealed the CO_2_ production rate, which was used to calculate TDEE considering assumptions about fractionation, dilution spaces and background levels of isotopes, and estimation of respiratory quotient^17^. Further, we used an average value of 0.85 to estimate the FQ in order to calculate the TDEE, which is based on conditions of energy balance and consuming an omnivore diet consumption^18,19^, both of which were present in our population.

To determine REE, we measured VCO_2_ production and VO_2_ consumption by indirect calorimetry in a respiration chamber^20^. Overnight-fasted participants entered the chamber in the morning of the second study center visit. We calculated REE based on the first 40 min of gas exchange after a 30-min equilibration phase and the individual nitrogen excretion rate (for calculation details see Supplementary Table S2) using established formulas^21^. Participants were instructed to refrain from sports, alcohol, caffeine, and eating after 9 pm the day before.

As DIT was estimated as 10% of TDEE^22^, AEE was calculated as 0.9 × TDEE minus REE.

The QUAP was developed at the German Cancer Research Center (DKFZ, Heidelberg, Germany) and considered several activity domains (i.e. occupation, domestic work and gardening, locomotion by foot and bicycle, leisure time activity spent with walking and bicycling, sports/exercise, sleeping/resting, sitting) and its yearly (summer vs. winter), monthly and weekly (weekday vs. weekend) variation^23^. The IPAQ-Short considered sitting, moderate, vigorous and walking activities of previous seven days across all domains^14^.

### Statistical analysis

For statistical analyses, we used SAS Enterprise Guide, version 4.3 (SAS Institute Inc., Cary, NC, USA). One participant was excluded because of abnormal thyroid hormone blood levels.

AEE prediction models were developed using linear stepwise regression analysis. Due to the high number of potential candidate variables (m = 75, after excluding missing-value variables) in contrast to 49 observations, we conducted a multi-step-selection process. In stage I, the 75 candidate variables were grouped by context or examination module, and within each group, significant variables were selected using stepwise regression on AEE with p-value limits of < 0.05 (stage I), that were considered for the next selection step (stage II).

The group of 15 accelerometer-derived variables was treated differently: each single variable was regressed on AEE to find out the most appropriate single parameter for AEE prediction. *VM counts* (33.8%) and *Axis 1 counts* (34.0%) explained the highest proportion of AEE variance. In this study, we focused only on *VM counts*, because it was frequently used in previous studies^13^, and revealed higher correlations in similar studies with larger sample sizes^24^.

In stage II, we developed final AEE prediction models using stepwise selection regression on all significant stage I variables with p-value limits < 0.05 (referred to as Model A).

In sensitivity analyses, we applied an additional stage II approach based on Schwarz Bayesian Information Criterion (SBC). Considering different combinations of p-value limits for including/retaining variables in the model [(a) 0.05/0.05, (b) 0.10/0.10, (c) 0.25/0.25, (d) 0.50/0.05, (e) 0.50/0.10, (f) 0.50/0.25], each model that emerged during one single step of the stepwise selection process that revealed a lower SBC compared to Model A was selected as Model B. While Models A contained only predictors that met the p-value threshold of < 0.05, Models B could include predictors that did not necessarily meet this threshold.

For practical application, various alternative models were developed at stage II simulating that only a reduced set of variables was available (e.g. due to not implemented measurements).

All selected models were checked for fulfilment of regression assumptions and collinearity. In sensitivity analyses, models were recalculated applying bootstrap sampling (2000 samples) at stage II.

## Results

We recruited 25 men and 25 women almost equally distributed over age and BMI groups (Supplementary Table S3). Participants were stable in weight during the 2-week examination period (mean weight difference 0.17 ± 0.95 kg). Table 1 shows the main characteristics for men and women; additional characteristics are presented in Supplementary Table S4. *FFM* (by ADP and BIA), *maximum handgrip strength*, *systolic blood pressure*, and *energy intake* were higher in men, while *relative fat mass* and *resting heart rate* were lower in men compared to women. Men had higher absolute TDEE, REE and AEE than women, whereas the relative proportions of REE and AEE were similar in both sexes. PA parameters derived from accelerometry and questionnaires (QUAP, IPAQ) were similar in men and women.

**Table 1:** Main characteristics of ActivE study population stratified by sex.

	Men (n = 25)			Women (n = 25)		
	Mean	± SD	(Min–Max)	Mean	± SD	(Min–Max)
**Age and anthropometry**						
Age (years)	49.9	± 13.8	(26.0–69.0)	40.0	± 14.6	(20.0–68.0)
Height (cm)	181.0	± 6.0	(172.1–194.1)	167.5	± 6.5	(156.8–183.6)
Weight (kg)	87.8	± 12.1	(67.0–120.1)	72.5	± 12.7	(52.4–97.2)
BMI (kg m^−2^)	26.8	± 3.5	(21.1–36.1)	25.9	± 4.6	(18.6–35.4)
FFM_ADP_ (kg)^a^	64.0	± 5.1	(53.6–72.2)	46.2	± 6.2	(34.3–60.5)
FM%_ADP_ (%)	26.3	± 8.3	(6.7–41.4)	35.0	± 10.8	(16.0–53.8)
**Fitness and circulatory parameters**						
Handgrip strength, maximum (kg)	49.0	± 7.1	(36.7–68.2)	31.5	± 7.3	(18.5–44.6)
Resting heart rate (bpm)	60.9	± 8.8	(43.5–80.5)	66.6	± 9.2	(52.0–85.0)
**Energy expenditure components**						
TDEE_DLW_ (kcal day^−1^)	3158	± 408	(2496–3905)	2571	± 464	(1813–3704)
REE_IC_ (kcal day^−1^)	1813	± 192	(1432–2256)	1501	± 141	(1273–1849)
AEE^b^ (kcal day^−1^)	1029	± 300	(342–1465)	813	± 339	(169–1611)
**Dietary data**						
Energy intake (kcal day^−1^)	2450	± 516	(1580–3890)	1957	± 496	(1190–3000)
Carbohydrate intake, relative (%)	43.5	± 7.1	(27.0–52.0)	47.2	± 6.9	(31.0–57.0)
**Accelerometry**^**c**^						
Vector magnitude counts (cpm)	439	± 126	(258–711)	458	± 119	(269–721)
Time in low PA (min day^−1^)	122	± 33	(71–196)	138	± 33	(83–242)
Time in moderate PA (min day^−1^)	97	± 28	(53–164)	100	± 21	(61–143)
Time in vigorous PA (min day^−1^)	22	± 11	(6–45)	21	± 12	(6–51)

	Median	(P25th, P75th)	Median	(P25th, P75th)		
**QUAP**						
Time in occupation (h week^−1^)	38.0	(0.0, 40.5)	39.0	(14.0, 40.0)		
Time in locomotion (h week^−1^)	2.5	(1.1, 4.5)	4.8	(1.8, 7.0)		
Time in domestic work/gardening (h week^−1^)	7.0	(5.0, 15.0)	10.0	(4.0, 15.0)		
Time in leisure time bicycling (h week^−1^)	0.6	(0.1, 1.4)	0.6	(0.1, 1.3)		
Time in leisure time walking (h week^−1^)	0.5	(0.2, 1.4)	1.0	(0.6, 1.4)		
Time in sports/exercise (h week^−1^)	2.7	(1.5, 5.5)	2.0	(1.2, 4.2)		

	Mean	± SD	(Min–Max)	Mean	± SD	(Min–Max)
Time in sedentary behavior (h day^−1^)	9.4	± 3.1	(2.6–14.0)	10.5	± 3.0	(3.7–16.1)

	Median	(P25th, P75th)	Median	(P25th, P75th)		
**IPAQ-short**						
Time in vigorous PA (min day^−1^)	25.7	(8.6, 42.9)	17.1	(4.3, 34.3)		
Time in moderate PA (min day^−1^)	5.7	(0.0, 25.7)	6.4	(0.0, 25.7)		
Time in walking activity (min day^−1^)	19.3	(4.3, 30.0)	17.1	(8.6, 60.0)		

	Mean	± SD	(Min–Max)	Mean	± SD	(Min–Max)
Time in sedentary behavior (h day^−1^)	7.9	± 3.3	(2.5–14.0)	6.9	± 3.8	(1.5–15.0)

Data are presented as mean, standard deviation (SD), minimum (Min) and maximum (Max), or as median, 25th and 75th percentile (P25th, P75th) separately for men and women. Additional characteristics are provided in Supplemental Digital Content Table 4.

^a^Similar results were obtained with the BIA method (see Supplemental Digital Content Table 4, which shows additional characteristics of ActivE study population stratified by sex).

^b^AEE was calculated as TDEE_DLW_ – REE_IC_ – DIT (assumed as 10% of TDEE).

^c^Intensity of physical activity was defined as low (79–2689 cpm), moderate (2690–6166 cpm), vigorous (6167 cpm and above) based on Vector magnitude counts per minute^15^.

*ADP* air-displacement plethysmography, *AEE* activity-related energy expenditure, *BMI* body mass index, *cpm* counts per minute, *DIT* diet-induced-thermogenesis, *DLW* doubly-labeled water, *FFM* fat-free mass, *FM* fat mass, *IC* indirect calorimetry, *IPAQ* International Physical Activity Questionnaire, *PA* physical activity, *QUAP* Questionnaire on Physical Activity on previous 12 months, *REE* resting energy expenditure, *TDEE* total daily energy expenditure.

### Stage I selection and crude analysis

Seventy-five candidate variables were assigned to 17 different groups, each including between 1 to 15 variables (Table 2). Group-wise stage I selection revealed a total of 11 variables significantly associated with AEE across groups (Table 2). Table 3 shows the crude association estimates and explained variances of each significant variable with AEE. *VM counts* was strongly positively associated with AEE and explained alone the largest variance in AEE (R^2^ = 33.8%), closely followed by *FFM*_*ADP*_ (R^2^ = 32.6%). *Time spent sitting*, *resting heart rate* and *sex* were inversely associated with AEE. Normal distribution and homogenous variance in the residuals were verified.

**Table 2:** Variable groups and its candidate variables after stage I selection using stepwise regression seperately for each group (n = 49).

Variable group	Candidate variables for stage I selection step	
	Offered to the model (groupwise)	Selected by the model (significant)
Accelerometry^a^	*(Separate analysis)*	VM counts^a^
ADP	FFM_ADP_, FM_ADP_, FM%_ADP_	FFM_ADP_
BIA	FFM_BIA_, FM_BIA_, FM%_BIA_	FFM_BIA_
Anthropometry	Height, weight, body mass index, waist circumference, hip circumference, waist-to-hip ratio, arm circumference	height
QUAP^b^	Time in occupation, MET-h in occupation, time in domestic work, MET-h in domestic work, time in bicycling, MET-h in bicycling, time in locomotion, time in walking, time in sports^b^	Time in locomotion
IPAQ	Time in vigorous PA, time in moderate PA, time in walking activity, total time in moderate and vigorous PA, total time in moderate PA and walking activity, total time in moderate and vigorous PA and walking	Total time in moderate PA and walking activity
Sitting (from QUAP & IPAQ)	Time spent sitting (IPAQ), time spent sitting (QUAP), time spent sitting on weekdays (QUAP), time spent sitting on weekend (QUAP)	Time spent sitting (QUAP)
Sleeping (from QUAP & IPAQ)	Time spent sleeping incl. napping (IPAQ), time spent sleeping incl. napping (QUAP), time spent sleeping excl. napping (QUAP)	*None*
Nutrition 1	Energy intake, fat intake, relative fat intake, carbohydrate intake, relative carbohydrate intake, protein intake, relative protein intake	Energy intake
Nutrition 2^c^	Carbohydrate intake, fat intake, relative fat intake, relative carbohydrate intake, protein intake, relative protein intake	Carbohydrate intake
Circulatory parameters	Systolic blood pressure, diastolic blood pressure, resting heart rate	Resting heart rate
Physical fitness	Handgrip strength	Handgrip strength
Demography	Age, sex	Sex
Metabolism	Fasting respiratory quotient	*None*
Socioeconomic	School education, professional qualification, occupation	*None*
Lifestyle	Smoking status, pack years of smoking, frequency of alcohol consumption, alcohol intake	*None*
Other	Season of examination	*None*

In each group all candidate variables were offered to select significant variables for AEE prediction by stepwise selection regression using p-value limits of 0.05 for the corresponding partial F-statistic for including and retaining variables in the model.

^a^In the accelerometry group, of 15 candidate variables the single parameter *VM counts* was selected because it explained the highest proportion of variance in AEE (33.8%, similar to *Axis 1 counts* 34.0%) using linear regression, was frequently used in previous studies^13^, and revealed higher correlations in similar designed studies with higher sample sizes^24^.

^b^In addition, we tested the following combined variables that were calculated as sum of the single variables: total time in locomotion, bicycling, walking, and sports; total time in locomotion, bicycling, walking, sports, and domestic work; total time in locomotion, bicycling, walking, sports, domestic work, and occupation.

^c^We considered two groups for nutrition: the second group does not include *energy intake*, as this variable is directly related to the aggregated intake of the macronutrients (carbohydrate, protein, and fat).

*ADP* air-displacement plethysmography, *BIA* bioelectrical impedance analysis, *FFM* fat-free mass, *FM* fat mass, *IPAQ* International Physical Activity Questionnaire, *MET* metabolic equivalent of task, *PA* physical activity, *QUAP* Questionnaire on Physical Activity on previous 12 months, *VM* vector magnitude.

**Table 3:** Crude association of significant variables from stage I selection step and AEE (kcal day^−1^) using single linear regression sorted by strength of association (n = 49).

Variables (stage I)	beta	SE	95% CI	p-value	STbeta	R^2^ (%)
VM counts (cpm)	1.60	0.33	(0.94, 2.26)	< 0.001	0.58	33.8
FFM_ADP_ (kg)	18.48	3.87	(10.69, 26.27)	< 0.001	0.57	32.6
FFM_BIA_ (kg)	15.29	3.96	(7.33, 23.25)	< 0.001	0.49	24.1
Height (cm)	18.07	4.71	(8.60, 27.53)	< 0.001	0.49	23.9
Energy intake (kcal d^−1)^	0.29	0.08	(0.14, 0.45)	< 0.001	0.48	23.2
Carbohydrate intake (g d^−1^)	2.08	0.62	(0.83, 3.33)	0.002	0.44	19.3
Sitting_QUAP_ (h day^−1^)	− 46.30	14.27	(− 75.00, − 17.60)	0.002	− 0.43	18.3
HGS_max_ (kg)	11.85	3.95	(3.90, 19.81)	0.004	0.40	16.0
Resting heart rate (bpm)	− 13.96	5.11	(− 24.24, − 3.69)	0.009	− 0.37	13.7
Locomotion_QUAP_ (h week^−1)^	21.40	8.78	(3.74, 39.06)	0.019	0.34	11.2
Sex (male = 0, female = 1)	− 220.05	92.14	(− 405.41, − 34.69)	0.021	− 0.33	10.8
MPA + walking_IPAQ_ (min day^−1^)	2.25	1.11	(0.01, 4.49)	0.049	0.28	8.0

*ADP* air-displacement plethysmography, *beta* unstandardized regression coefficient, *BIA* bioelectrical impedance analysis, *bpm* beats per minute, *cpm* counts per minute, *FFM* fat-free mass, *FM* fat mass, *HGS* handgrip strength, *IPAQ* International Physical Activity Questionnaire, *MPA* moderate physical activity, *QUAP* Questionnaire on Physical Activity on previous 12 months, *R*^*2*^ coefficient of determination, *SE* standard error, *STbeta* standardized beta coefficient, *VM* vector magnitude.

### Stage II selection for final AEE prediction models

Table 4 presents the results of the stage II stepwise selection process for the AEE prediction models. We used the term ‘selected’ for variables that were offered for stepwise regression, and were statistically selected based on the p < 0.05 threshold. When all 11 significant variables from stage I were considered for stage II selection step, *VM counts, FFM*_*ADP*_, *time in moderate PA and walking*, and *carbohydrate intake* were selected as predictor variables, which explained 70.7% of the AEE variance.

**Table 4:**
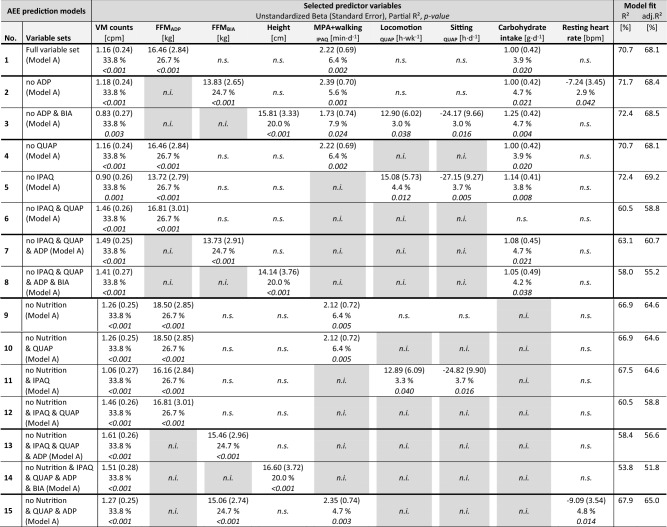
AEE prediction models derived from stage II stepwise selection regression using accelerometer variable VM counts and full or reduced variable sets (n = 49).

Predictors were selected applying stepwise regression with p-value limits of 0.05 (Model A). Gray-shaded variables were not offered for selection in the specific setting. More details and the results of the sensitivity analyses revealing additional models are shown in Supplemental Digital Content Table 5 (Details of prediction models for AEE [kcal day^−1^] derived from stepwise selection regression using accelerometer-derived VM counts and full or reduced sets of preselected stage I variables).

*AEE* activity-related energy expenditure, *ADP* air-displacement plethysmography, *BIA* bioelectrical impedance analysis, *bpm* beats per minute, *cpm* counts per minute, *FFM* fat-free mass, *IPAQ* International Physical Activity Questionnaire, *MPA* moderate physical activity, *n.i.* not included (not offered for selection step), *n.s.* not selected, *QUAP* Questionnaire on Physical Activity on previous 12 months, *VM* vector magnitude.

Running the stage II selection step with reduced sets of stage I variables offered for stepwise regression, we examined to what extent other variables may substitute in explaining AEE variance (Table 4, Supplementary Table S5). In all these models, *VM counts* (considered in every model) was always selected as first predictor, and *FFM*_*ADP*_ (when considered) was always selected as second predictor. *Time in moderate PA and walking* derived from IPAQ was always selected as predictor, if information from IPAQ was considered (Table 4).

If no ADP to estimate FFM was considered, then *FFM*_*BIA*_ was selected instead (Table 4). If no information from ADP or BIA was considered at all, then *height* was selected instead.

If no information from IPAQ was considered, then *time spent sitting* and *time in locomotion* derived from QUAP were selected instead (if considered). If no information from neither QUAP nor IPAQ was considered, no other predictor was selected instead.

*Carbohydrate intake* was selected in almost every model, if information on nutrition was considered; otherwise no other predictor was selected instead. *Resting heart rate* was selected only in a few models. The variables *sex*, *handgrip strength*, and *energy intake* were not selected in any AEE prediction model.

Across the examined variable sets, 53.8–72.4% of the variance in AEE was explained by 2–6 predictors included in the models (Table 4). *VM counts* and *FFM* (by ADP or BIA) or alternatively *height* contributed most to the explained AEE variance (partial R^2^: *VM counts* = 33.8%, *FFM*_*ADP*_ = 26.7%, *FFM*_*BIA*_ = 24.7%, *height* = 20.0%). The other predictors—when selected—contributed a smaller proportion to the explained AEE variance (partial R^2^ range: 2.9–7.9%).

### Application and sensitivity analyses

**Figure 1:**
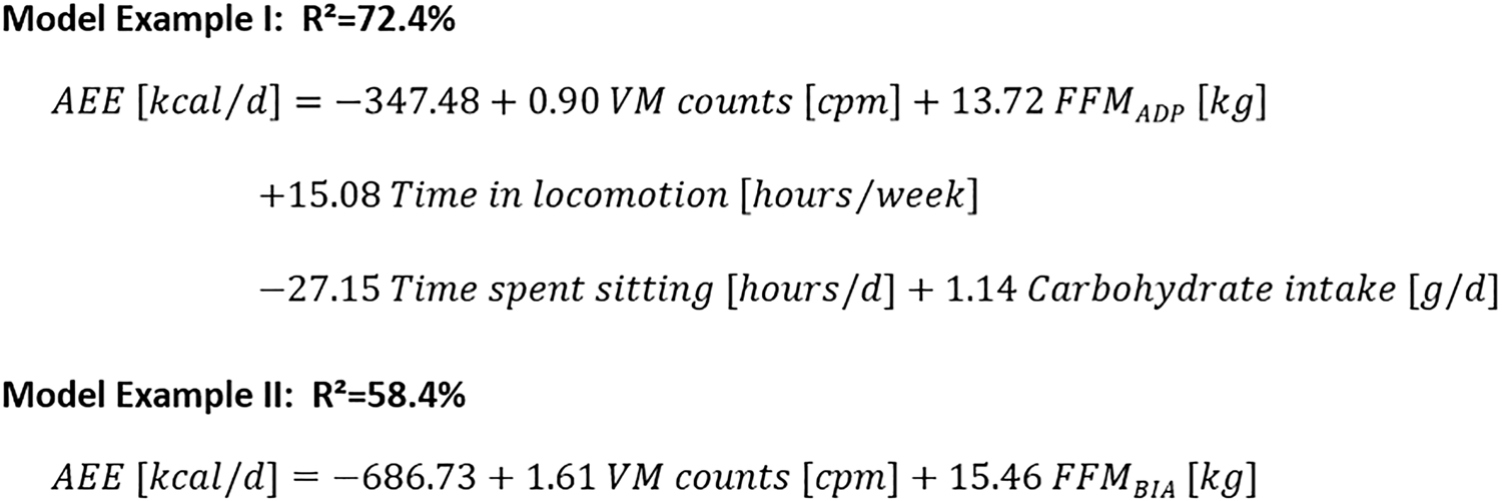
Examples of AEE prediction models when information about accelerometry, ADP, QUAP and nutrition is available (Example I, Table 4 model No. 5), and when only information about accelerometry and BIA is available (Example II, Table 4 model No. 13). More details in Supplementary Table S5. *ADP* air-displacement plethysmography, *AEE* activity-related energy expenditure, *BIA* bioelectrical impedance analysis, *FFM* fat-free mass, *cpm* counts per minute, *PA* physical activity, *VM* vector magnitudes.

The results of our analysis may easily be used to predict AEE depending on the availability of variables (Table 4, and all necessary details for calculation in Supplemental Digital Content Table 5). In Fig. 1, Model Example I illustrates the application of the prediction equation for the model with the highest explained variance (Model No.5 from Table 4, for calculation details see also Supplemental Digital Content Table 5). In this example, a person with the following characteristics, *VM counts* = 440 cpm, *FFM*_*ADP*_ = 55 kg, *FFM*_*BIA*_ = 55 kg, *time in locomotion* = 3.5 h/week*, time spent sitting* = 10 h/day*, carbohydrate intake* = 250 g/day, would have a predicted AEE of 869 kcal/day. In Fig. 1, Model Example II illustrates the application if only a minimal variable set is available. In this example, when information only on accelerometry and BIA is available (Model No.13 from Table 4, for calculation details see also Supplemental Digital Content Table 5), the same person would have a predicted AEE of 872 kcal/day.

In sensitivity analyses, when selection was based on improved SBC, additional AEE prediction models were developed in five variable settings (listed as Model B in Supplementary Table S5). In the full variable set, 75.4% of AEE variance was explained by the predictors *VM counts* (33.8%), *FFM*_*ADP*_ (26.7%), *time in moderate PA and walking* (6.4%), *time in locomotion* (2.4%), *time spent sitting* (2.3%) and *carbohydrate intake* (3.9%).

When AEE prediction models were recalculated using bootstrap sampling and stepwise regression, the bootstrap inclusion frequencies of the selected variables reflected the results obtained from original analysis.

All AEE prediction models met the assumptions of normally distributed residuals and homoscedasticity, and no substantial multi-collinearity among the predictors was detected.

## Discussion

In this study, we developed models to predict free-living AEE based on accelerometry information and additional predictors. Considering a variety of potential variables, we found a model that included *VM counts*, *FFM*_*ADP*_, *time in moderate PA and walking (IPAQ)* and *carbohydrate intake* explaining 70.7% of variance in free-living AEE. Considering reduced sets of variables, we developed models explaining 53.8–72.4% of variance in free-living AEE depending on the number of selected predictors. Models developed in reduced variable sets could explain a higher AEE variance compared to the ‘full variable set’ model, if unavailable variables were substituted by other or a higher number of predictors. The results of our analysis may be used in studies using accelerometry for AEE prediction.

The triaxial accelerometer output *VM counts* explained 33.8% of the variance in AEE, which is in the range (22.0–49.0%) of previous studies^25–29^. The anthropometric variables (*FFM*_*ADP*_*, FFM*_*BIA*_*, height*) added the highest proportion of explained AEE variance (20.0–26.7%) in addition to *VM counts*. Other selected predictors (*time in moderate PA and walking*, *time spent sitting*, *time in locomotion*, *carbohydrate intake*, *resting heart rate*) contributed a much lower proportion ranging from 2.9 to 7.9%.

These findings emphasize the relevance of anthropometric variables for AEE prediction in addition to objective PA parameters as derived from accelerometry. Two-parameter models, comprised of *VM counts* and *FFM* or *height*, explained 53.8–60.5% of AEE variance, which was also found in previous studies^27,30^. Interestingly, *FFM* and *height* were never included together in a model^27,30–34^, probably due to the strong relation between these variables (based on their relation to sex), that would have induced multi-collinearity problems. Physiologically plausible, *FFM* reflects the skeletal muscle mass that represents the major location of energy expenditure due to PA movement^1,3^. Similarly, *height* reflects the sex-based differences in *FFM*. Some previous studies included *height* in combination with *body weight*^32,34^, which comprises *fat mass* and *FFM*. However, in this study, *weight* was not selected during the stage I selection step of the anthropometry variable group, and was therefore not offered for stage II selection to develop AEE prediction models.

Self-reported PA information from questionnaires contributed slightly to moderately to AEE variance. These parameters may include relevant information about activities that were not adequately captured by the accelerometer, such as cycling, static exercises, or water activities^4^. Therefore additional PA information from questionnaires could further improve AEE prediction, although the effort to obtain this information and its validity has to be considered^4,8^.

Interestingly, even the inversely associated variable *time spent sitting* contributed to AEE variance, although it rather reflects inactivity without substantial energy expenditure. This is, to the best of our knowledge, the first study using questionnaire-based PA parameters for DLW-derived AEE prediction.

Surprisingly, *carbohydrate intake* contributed to AEE variance, although this contribution was rather small. We suppose that additional information on PA-related attributes might be covered in this variable, such as that more active people differ in their diet regarding carbohydrate composition compared to less active people^35^. However, the effort to obtain nutritional information was disproportionate to the improvement of explained variance in this study. Further, nutritional information is prone to underreporting, which was also observed in this study. Therefore, results on nutritional aspects should be interpreted cautiously.

The relevance of *resting heart rate* in AEE prediction might be questionable, because, if selected, it contributed only marginally to AEE variance. Nevertheless, it is an easy-to-measure parameter and might indicate overall fitness levels with lower heart rates in fitter people^36^. Unfortunately, we could not find studies using *resting heart rate* for DLW-derived AEE prediction.

The variables *sex, handgrip strength* and *energy intake* were not selected for AEE prediction during stage II selection step, although they showed moderate associations in the univariate models. We suppose that the inclusion of *FFM* or *height* already reflected these associations, that is based on the strong association of *sex* with *FFM*, *height*^37^, *handgrip strength*^38^, and *energy intake* (as surrogate for TDEE)^39,40^. In comparable studies using stepwise selection regression, *sex* was not selected when *height* or *FFM* was included in the model^27,30^.

The main strengths of this study were the sophisticated reference method (DLW), and the large number of potential candidate variables to predict AEE, most of which were considered for the first time. Furthermore, the grouping of variables by examination module or context enabled AEE prediction in different variable settings, allowing to select appropriate models depending on the availability of predictors. For example, in the German National Cohort study, information on 7-day-accelerometry, BIA, anthropometry, and GPAQ^41^ (which is similar to IPAQ^14^) were collected from a large population-based sample^6^. Some participants had additional PA information from QUAP, or nutritional information from food-frequency-questionnaires^6^. Thus, the developed AEE prediction models could be applied to this sample to obtain AEE estimates to further investigate associations with biomarker concentrations or chronic disease risks.

Another important strength is the 24-h assessment of free-living PA by accelerometry for 2 weeks that provides a reliable estimate of habitual PA in adults^42^. Both the measurement of PA by accelerometry and the measurement of TDEE by DLW method were performed simultaneously, so that recorded PA should be reflected in the calculated AEE. Non-wear times of the accelerometer during waking hours amounted to about 15 min per day (median) according to wearing diary. This is very low and therefore not to be expected to influence the accelerometer-derived PA outcome.

Further, the ActivE study population covered a large range of age and BMI for both sexes, which creates more heterogeneity and variability in the data to promote generalizability of study findings. With 49 participants, as determined from sample size calculation, the analytic sample size was adequate and comparable to previous studies of similar study design and aim^27,29,43^.

On the other hand, one main limitation is the imbalance between the number of candidate variables and observations. To overcome this, first, we applied conceptual and statistical (pre)-selection of candidate variables. Second, we chose rigorous p-value limits (p < 0.05) for stepwise selection regression to promote selection of variables with stronger effects and to prevent overfitting^44^. Third, in sensitivity analyses using bootstrap sampling, the selected predictors were confirmed.

Another main limitation is that the developed prediction models were not externally validated. Further, the sample size of our study is rather small—despite statistically adequate–, and the population includes only healthy volunteers. Thus, the population is not representative, and transferring our results to the general population or other specific groups must be conducted with caution.

Another limitation is the use of the proprietary accelerometer output ‘activity counts’ provided by the software, which generally differs across monitors and manufacturers, and therefore lacks comparability^8,45,46^. Thus, the developed prediction models are basically limited to ActiGraph’s® GT3X + derived activity counts. Measurements from other devices would have to be validated before using the prediction models. Ideally, a comparable measure, such as raw acceleration data, should be used for model development, whereby all filter and processing steps of the acceleration signals should be considered in a transparent and comprehensible way. This will increase the complexity of the analysis^47^, but it will likely improve the comparability between future studies in instances where different devices were used, and would thereby potentially allow for a broader application of our developed prediction models.

Further limiting is that one accelerometer placed at the hip cannot capture all human activities properly^4, 8^. Consequently, an inaccurate assessment of some activities could lead to underestimation of PA and might have attenuated the association to energy expenditure^8^.

## Conclusion

We developed models to predict free-living AEE based on accelerometry information and various additional predictors. Considering all potential variables, we found a model that included *VM counts*, *FFM*_*ADP*_, *time in moderate PA and walking,* and *carbohydrate intake*, and explained 70.7% of variance in free-living AEE. AEE prediction models developed in reduced sets of available variables explained 53.8–72.4% of AEE variance, depending on the number of selected predictors. The results of our analysis may be used for AEE prediction in studies using accelerometry.

## Supplementary Information

Supplementary Information.

## Data Availability

The data that support the findings of this study are available from the authors but restrictions apply to the availability of these data, and so are not publicly available. Interested researchers (who meet criteria for access to confidential data) may contact the corresponding author of our manuscript for access to the datasets generated or analyzed during the current study.
