# Supplementary material for: Prediction of activity-related energy expenditure under free-living conditions using accelerometer-derived physical activity
Source: Sci Rep. 2022 Oct 4;12:16578. doi: 10.1038/s41598-022-20639-0 (PMC9532429; doi:10.1038/s41598-022-20639-0)
Supplement: Supplementary file 1 — Supplementary Information. [file 41598_2022_20639_MOESM1_ESM.pdf]

Supplementary information

Article title:

Prediction of activity-related energy expenditure under free-living conditions using accelerometer-derived physical activity

Author names:

Stephanie Jeran, Astrid Steinbrecher, Verena Haas, Anja Mähler, Michael Boschmann, Klaas R. Westerterp, Boris A. Brühmann, Karen Steindorf, Tobias Pischon

Corresponding author:

Tobias Pischon, Molecular Epidemiology Research Group, Max Delbrück Center for Molecular Medicine (MDC) in the Helmholtz Association, Robert-Rössle-Straße 10, 13125 Berlin, Germany, Phone: ++49 30/9406-4563, E-mail: tobias.pischon@mdc-berlin.de

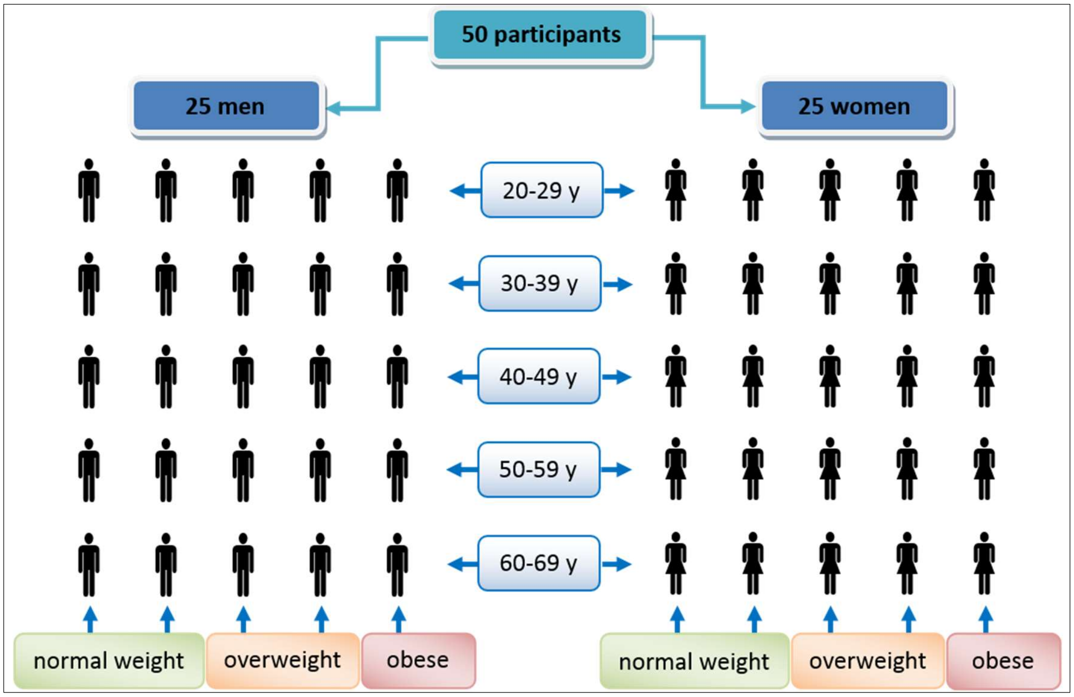

Supplementary Fig.1 Recruitment scheme of target sampling

Supplementary Table 1 Results of non-wear time (NWT) analysis of accelerometer data using ActiLife software in six participants with detected NWT periods of critical length

**Total NWT period** [hours:minutes]	**Reason for taking accelerometer off** (information obtained from diary)	**NWT during waking phase**[a] [hours]	**Decision of NWT treatment**
18h22m	delayed change of accelerometer for 2nd week	11h	NWT was considered by deleting that complete day from further calculation
16h39m	showering, forget to put accelerometer on again	7h (3.5h/day)[b]	NWT (per day) not relevant
11h	no reason specified	3h	NWT not relevant
10h57m	forget to put accelerometer on again	2h	NWT not relevant
8h15m	sauna visit	8h	NWT during activity of low intensity (not considered)
7h6m	delayed change of accelerometer for 2nd week	<1h	NWT not relevant

The used NWT algorithm (2 hours of consecutive zeros in VM counts, interruptions were not allowed) detected 26 participants without any NWT, 7 participants having NWT during sleeping at night (verified with accelerometer diary), which was not considered as NWT, and 17 participants having NWT during waking hours or during sleeping and waking hours. Of these, NWT of 11 participants* amounted to 2 – 3 hours per day, which seemed not relevant in relation to a 24-hour day, so these NWT were not considered. Of the remaining 6 participants – listed in the table– with detected NWT between 7 – 18 hours (after verification with diary), only 1 participant had relevant NWT during waking hours, which was considered by deleting the complete day from the calculation of the accelerometer parameters.

[a] If NWT includes sleeping phase (verified with diary) this time was deducted, because NWT during sleeping was not considered as relevant for PA measurement.

[b] NWT during waking hours was evenly distributed over 2 days (before and after midnight).

* According to the diary, reasons for NWT were: sauna visits (3 participants), sport with high body contact and taking a shower (2 participants), taking a shower/bath (2 participants), swimming (1 participant), changing clothes without accidentally putting the accelerometer on again (1 participant); and no specified reason (2 participants)

NWT non-wear time

Supplementary Table 2 Formulas for step-by-step calculation of the individual nitrogen (N) excretion rate (used for the calculation of resting energy expenditure)

**Calculation of individual nitrogen excretion rate**

Sum of excreted N [mg/ml] from urea, ammonium, creatinine, uric acid	=	*(Urea [mol/l] * 28.0134 g N/mol)* + *(Ammonium [mol/l] * 14.0067 g N/mol)* + *(Creatinine [mol/l] * 42.0201 g N/mol)* + *(Uric acid [mol/l] * 56.0268 g N/mol)*	(Eq. 1)

Urine volume of whole voiding [ml]	=	*Urine mass of whole voiding [g]* / *Specific weight of urine sample [g/ml]*	(Eq. 2)

Time interval [min] for resting phase	=	*Time of voiding before REE measurement* − *Time of preceding voiding (at home)*	(Eq. 3)

Amount of excreted N [mg] of REE urine sample	=	*Sum of excreted N [mg/ml] * Urine volume [ml]*	(Eq. 4)

N excretion rate [mg/min] for resting phase	=	*Amount of excreted N [mg]* / *Time interval for resting phase [min]*	(Eq. 5)

Nitrogen excretion rate for the resting phase was estimated by measuring the concentration of the nitrogen-containing compounds urea, ammonia/ammonium, creatinine, and uric acid in the urine sample that was collected immediately before entering the respiration chamber (which also corresponds to the second voiding of the day), and that covers the fasting period from getting up in the morning until entering the chamber. From the measured concentration of the nitrogen containing compounds, the excreted nitrogen (N) of each compound was calculated and summed up (Eq. 1). The volume of the urine sample was calculated from measured urine mass (mass of the whole voiding) and measured specific weight of the urine sample (Eq. 2). Considering the times of urine sample collection (Eq. 3), and the amount of excreted nitrogen per urine sample (Eq. 4), finally the nitrogen excretion rate for the resting phase was calculated (Eq. 5). We assume that the resting nitrogen excretion rate, which practically was obtained immediately before the REE measurement, also corresponds during the actual REE measurement.

Supplementary Table 3 Number of ActivE study participants stratified by sex, BMI, and age group

Age group	Men (n=25)				Women (n=25)			
	Total	BMI 18.5-24.9	BMI 25.0-29.9	BMI ≥30.0	Total	BMI 18.5-24.9	BMI 25.0-29.9	BMI ≥30.0
20-29 years	2	2	-	-	8	4	3	1
30-39 years	5	2	2	1	5	3	2	-
40-49 years	6	1	4	1	5	2	2	1
50-59 years	4	1	2	1	5	2	1	2
60-69 years	8	3	4	1	2	-	2	-
Total	25	9	12	4	25	11	10	4

Supplementary Table 4 Additional characteristics of ActivE study population stratified by sex

	Men (n=25)			Women (n=25)		
	**Mean**	**± SD**	**(Min - Max)**	**Mean**	**± SD**	**(Min - Max)**
Anthropometry						
FFM_{BIA} [kg]	64.6	± 6.1	(53.5 - 79.7)	46.6	± 6.0	(36.7 - 58.4)
$FM\%_{BIA}$ [%]	25.9	± 6.5	(9.3 - 37.8)	34.8	± 8.0	(24.4 - 48.9)
Fitness & Circulatory parameters						
Systolic blood pressure [mmHg]	126.6	± 10.4	(110.5 - 149.5)	117.4	± 15.2	(99.0 - 157.5)
Diastolic blood pressure [mmHg]	78.9	± 6.3	(69.0 - 93.5)	74.2	± 9.8	(57.5 - 96.0)
Dietary data						
Fat intake, relative [%]	33.6	± 4.8	(27.0 - 49.0)	31.7	± 6.0	(19.0 - 44.0)
Protein intake, relative [%]	16.6	± 2.5	(12.0 - 22.0)	16.2	± 3.2	(11.0 - 26.0)
Accelerometry [a]						
Time in inactivity [min/d]	1199	± 63	(1061 - 1293)	1181	± 53	(1042 - 1278)
QUAP						
Time spent sleeping [h/night]	7.0	± 0.7	(5.5 - 8.0)	7.3	± 0.9	(5.5 - 9.0)
Time spent sleeping incl. napping [h/d]	7.2	± 0.7	(6.0 - 8.5)	7.5	± 1.1	(5.5 - 11.0)
IPAQ-Short						
Time spent sleeping [h/d]	7.3	± 1.0	(5.0 - 10.0)	7.1	± 1.0	(5.0 - 10.0)
	**N**	**(%)**		**N**	**(%)**	
Socioeconomic status & Lifestyle						
**Occupation**						
Full-time employed	14	(56 %)		14	(56 %)	
Part-time employed[b]	2	(8 %)		8	(32 %)	
Not employed	9	(36 %)		3	(12 %)	
**School education**						
No qualification for university entrance	7	(28 %)		4	(16 %)	
Qualification for university entrance	18	(72 %)		21	(84 %)	
**Professional qualification**						
Completed vocational / educational training	8	(32 %)		7	(28 %)	
Completed academical training	9	(36 %)		10	(40 %)	
Completed both trainings	8	(32 %)		8	(32 %)	
**Alcohol consumption, frequency**						
1x per week or less	12	(48 %)		16	(64 %)	
More than 1x per week	13	(52 %)		9	(36 %)	
**Smoking status**						
Never smoker	9	(36 %)		12	(48 %)	
Current smoker	7	(28 %)		5	(20 %)	
Former smoker	9	(36 %)		8	(32 %)	
**Season of examination**						
Spring	5	(20 %)		6	(24 %)	
Summer	3	(12 %)		2	(8 %)	
Autumn	9	(36 %)		9	(36 %)	

Winter	8	(32 %)	8	(32 %)

Data are presented as mean, standard deviation (SD), minimum (Min) and maximum (Max), or as absolute (N) and relative numbers (%) separately for men and women.

[a] Intensity of physical activity was defined as inactive (0-78 cpm), low (79-2689 cpm), moderate (2690-6166 cpm), vigorous (6167 cpm and above) based on Vector magnitude counts per minute [15].

[b] part-time employed includes also the categories semi-retirement, marginally / irregularly / occasionally employed

BIA bioelectrical impedance analysis, cpm counts per minute, FFM fat-free mass, FM fat mass, IPAQ International Physical Activity Questionnaire, QUAP Questionnaire on Physical Activity on previous 12 months.

Supplementary Table 5 Details of prediction models for AEE [kcal/d] derived from stepwise selection regression using accelerometer-derived VM counts and full or reduced sets of preselected stage I variables (n=49)

Setting Model	Selected predictor	beta	SE	p-value	STbeta	pR²	R²	adj.R²	SBC
full variable set									
Model A	Intercept	-883.32	179.88	<0.001	0.00	.	0.707	0.681	528.89
	VM counts [cpm]	1.16	0.24	<0.001	0.42	0.338			
	FFM_{ADP} [kg]	16.46	2.84	<0.001	0.51	0.267			
	MPA+$walking_{IPAQ}$ [min/d]	2.22	0.69	0.002	0.28	0.064			
	Carbohydrate intake [g/d]	1.00	0.42	0.020	0.21	0.039			
Model B	Intercept	-515.50	226.21	0.028	0.00	.	0.754	0.719	528.06
	VM counts [cpm]	0.85	0.25	0.001	0.31	0.338			
	FFM_{ADP} [kg]	15.05	2.73	<0.001	0.47	0.267			
	MPA+$walking_{IPAQ}$ [min/d]	1.55	0.69	0.029	0.20	0.064			
	Carbohydrate intake [g/d]	1.15	0.39	0.006	0.24	0.039			
	$Sitting_{QUAP}$ [h/d]	-21.63	9.18	0.023	-0.20	0.023			
	$Locomotion_{QUAP}$ [h/week]	11.56	5.69	0.048	0.18	0.024			
no ADP									
Model A	Intercept	-304.02	334.71	0.369	0.00	.	0.717	0.684	531.17
	VM counts [cpm]	1.18	0.24	<0.001	0.43	0.338			
	FFM_{BIA} [kg]	13.83	2.65	<0.001	0.44	0.247			
	Carbohydrate intake [g/d]	1.00	0.42	0.021	0.21	0.047			
	MPA+$walking_{IPAQ}$ [min/d]	2.39	0.70	0.001	0.30	0.056			
	Resting heart rate [bpm]	-7.24	3.45	0.042	-0.19	0.029			
Model B	Intercept	-498.07	232.75	0.038	0.00	.	0.743	0.706	530.35
	VM counts [cpm]	0.97	0.26	<0.001	0.35	0.338			
	FFM_{BIA} [kg]	13.44	2.58	<0.001	0.43	0.247			
	Carbohydrate intake [g/d]	1.33	0.40	0.002	0.28	0.047			
	MPA+$walking_{IPAQ}$ [min/d]	1.29	0.69	0.070	0.16	0.056			
	$Sitting_{QUAP}$ [h/d]	-23.64	9.33	0.015	-0.22	0.029			
	$Locomotion_{QUAP}$ [h/week]	12.08	5.82	0.044	0.19	0.026			
no ADP & BIA									
Model A	Intercept	-2442.23	604.51	<0.001	0.00	.	0.724	0.685	533.73
	VM counts [cpm]	0.83	0.27	0.003	0.30	0.338			
	Height [cm]	15.81	3.33	<0.001	0.43	0.200			
	MPA+$walking_{IPAQ}$ [min/d]	1.73	0.74	0.024	0.22	0.079			
	Carbohydrate intake [g/d]	1.25	0.42	0.004	0.26	0.047			
	$Sitting_{QUAP}$ [h/d]	-24.17	9.66	0.016	-0.22	0.030			
	$Locomotion_{QUAP}$ [h/week]	12.90	6.02	0.038	0.20	0.030			
no QUAP									
Model A	Intercept	-883.32	179.88	<0.001	0.00	.	0.707	0.681	528.89
	VM counts [cpm]	1.16	0.24	<0.001	0.42	0.338			
	FFM_{ADP} [kg]	16.46	2.84	<0.001	0.51	0.267			
	MPA+$walking_{IPAQ}$ [min/d]	2.22	0.69	0.002	0.28	0.064			
	Carbohydrate intake [g/d]	1.00	0.42	0.020	0.21	0.039			

Supplementary Table 5 *Continued*

Setting Model	Selected predictor	beta	SE	p-value	STbeta	pR²	R²	adj.R²	SBC
no IPAQ									
Model A	Intercept	-347.48	223.70	0.128	0.00	.	0.724	0.692	529.81
	VM counts [cpm]	0.90	0.26	0.001	0.33	0.338			
	FFM_{ADP} [kg]	13.72	2.79	<0.001	0.42	0.267			
	$Sitting_{QUAP}$ [h/d]	-27.15	9.27	0.005	-0.25	0.037			
	Carbohydrate intake [g/d]	1.14	0.41	0.008	0.24	0.038			
	$Locomotion_{QUAP}$ [h/week]	15.08	5.73	0.012	0.24	0.044			
no IPAQ & QUAP									
Model A	Intercept	-685.55	195.88	0.001	0.00	.	0.605	0.588	535.78
	VM counts [cpm]	1.46	0.26	<0.001	0.53	0.338			
	FFM_{ADP} [kg]	16.81	3.01	<0.001	0.52	0.267			
Model B	Intercept	-763.66	193.78	<0.001	0.00	.	0.637	0.613	535.50
	VM counts [cpm]	1.37	0.25	<0.001	0.50	0.338			
	FFM_{ADP} [kg]	14.87	3.08	<0.001	0.46	0.267			
	Carbohydrate intake [g/d]	0.91	0.46	0.052	0.19	0.032			
no IPAQ & QUAP & ADP									
Model A	Intercept	-800.80	202.20	<0.001	0.00	.	0.631	0.607	536.28
	VM counts [cpm]	1.49	0.25	<0.001	0.54	0.338			
	FFM_{BIA} [kg]	13.73	2.91	<0.001	0.44	0.247			
	Carbohydrate intake [g/d]	1.08	0.45	0.021	0.23	0.047			
no IPAQ & QUAP & ADP & BIA									
Model A	Intercept	-2453.02	636.57	<0.001	0.00	.	0.580	0.552	542.62
	VM counts [cpm]	1.41	0.27	<0.001	0.51	0.338			
	Height [cm]	14.14	3.76	<0.001	0.38	0.200			
	Carbohydrate intake [g/d]	1.05	0.49	0.038	0.22	0.042			
no Nutrition / no Nutrition & QUAP									
Model A	Intercept	-792.23	184.99	<0.001	0.00	.	0.669	0.646	531.06
	VM counts [cpm]	1.26	0.25	<0.001	0.46	0.338			
	FFM_{ADP} [kg]	18.50	2.85	<0.001	0.57	0.267			
	$MPA+walking_{IPAQ}$ [min/d]	2.12	0.72	0.005	0.27	0.064			
Model B	Intercept	-235.91	342.11	0.494	0.00	.	0.694	0.666	531.04
	VM counts [cpm]	1.14	0.25	<0.001	0.42	0.338			
	FFM_{ADP} [kg]	16.85	2.90	<0.001	0.52	0.267			
	$MPA+walking_{IPAQ}$ [min/d]	2.49	0.73	0.001	0.31	0.064			
	Resting heart rate [bpm]	-6.78	3.55	0.062	-0.18	0.025			
no Nutrition & IPAQ									
Model A	Intercept	-288.06	238.88	0.234	0.00	.	0.675	0.646	533.94
	VM counts [cpm]	1.06	0.27	<0.001	0.39	0.338			
	FFM_{ADP} [kg]	16.16	2.84	<0.001	0.50	0.267			
	$Sitting_{QUAP}$ [h/d]	-24.82	9.90	0.016	-0.23	0.037			
	$Locomotion_{QUAP}$ [h/week]	12.89	6.09	0.040	0.20	0.033			
no Nutrition & IPAQ & QUAP									
Model A	Intercept	-685.55	195.88	0.001	0.00	.	0.605	0.588	535.78
	VM counts [cpm]	1.46	0.26	<0.001	0.53	0.338			
	FFM_{ADP} [kg]	16.81	3.01	<0.001	0.52	0.267			

Supplementary Table 5 *Continued*

Setting Model	Selected predictor	beta	SE	p-value	STbeta	pR^2	R^2	adj.R^2	SBC
no Nutrition & IPAQ & QUAP & ADP									
Model A	Intercept	-686.73	206.45	0.002	0.00	.	0.584	0.566	538.29
	VM counts [cpm]	1.61	0.26	<0.001	0.59	0.338			
	FFM_{BIA} [kg]	15.46	2.96	<0.001	0.50	0.247			
no Nutrition & IPAQ & QUAP & ADP & BIA									
Model A	Intercept	-2672.31	652.01	<0.001	0.00	.	0.538	0.518	543.45
	VM counts [cpm]	1.51	0.28	<0.001	0.55	0.338			
	Height [cm]	16.60	3.72	<0.001	0.45	0.200			
no Nutrition & QUAP & ADP									
Model A	Intercept	-48.35	333.58	0.885	0.00	.	0.679	0.650	533.38
	VM counts [cpm]	1.27	0.25	<0.001	0.46	0.338			
	FFM_{BIA} [kg]	15.06	2.74	<0.001	0.48	0.247			
	MPA+$walking_{IPAQ}$ [min/d]	2.35	0.74	0.003	0.30	0.047			
	Resting heart rate [bpm]	-9.09	3.54	0.014	-0.24	0.048			

In Model A predictors were selected using stepwise-selection regression with p-value limits of <0.05 for the partial F-statistic for including and retaining variables in the model. Model B was selected if during one step of the stepwise selection process where six p-value combinations were applied for the partial F-statistic for including/retaining variables [(a) 0.05/0.05, (b) 0.10/0.10, (c) 0.25/0.25, (d) 0.50/0.05, (e) 0.50/0.10, (f) 0.50/0.25], the included variables revealed a lower SBC compared to Model A (results of sensitivity analyses).

adj. adjusted, ADP air-displacement plethysmography, beta unstandardized regression coefficient, BIA bioelectrical impedance analysis, bpm beats per minute, cpm counts per minute, FFM fat-free mass, IPAQ International Physical Activity Questionnaire, MPA moderate physical activity, QUAP Questionnaire on Physical Activity on previous 12 months, SE standard error of beta, STbeta standardized regression coefficient, pR^2 partial explained variance of the predictor, R^2 explained variance of the model, SBC Schwarz Bayesian Criterion, VM vector magnitude.
